# The Application of Pinch Technology to a Novel Closed-Loop Spray Drying System with a Condenser and Reheater

**DOI:** 10.3390/e26090809

**Published:** 2024-09-23

**Authors:** Zexin Lei, Thomas O’Neill, Timothy Langrish

**Affiliations:** Drying and Process Technology Group, School of Chemical and Biomolecular Engineering, Building J01, The University of Sydney, Darlington, NSW 2006, Australia; tone0373@uni.sydney.edu.au (T.O.); timothy.langrish@sydney.edu.au (T.L.)

**Keywords:** pinch analysis, closed-loop spray-drying system, air reheater, heat recovery, approach temperatures

## Abstract

Spray drying is an energy-intensive process in industrial use, making energy recovery a critical focus for improving overall efficiency. This study investigates the potential of integrating heat-recovery systems, including an innovative air reheater, into a closed-loop spray-drying unit to maximise energy savings. Through detailed pinch analysis, the system achieved a very low approach temperature, averaging 3.48 K, which is significantly lower than that of conventional open-loop systems. The study quantifies the energy-recovery potential by demonstrating that the integration of heat-recovery components can reduce the external heating demand by up to 30%. This not only enhances heat-transfer efficiency but also lowers operational costs and reduces the system’s environmental impact. The results suggest that closed-loop systems with air reheaters offer a scalable solution for improving energy efficiency across different industrial applications. The research highlights a new paradigm: focusing on latent energy within the system rather than adjusting individual operational variables.

## 1. Introduction

A drying system separates liquid or moisture from a substance through controlled heat, mass, and momentum transfer [1]. Drying preserves the dried materials and reduces product volume and weight, facilitating storage and handling. It is a traditional preservation method, extending back thousands of years [2], now utilized across various industries, such as industries producing timber, polymers, ceramics, minerals, and pharmaceuticals [3]. Spray dryers are examples of dryers that are used to produce powders from solutions or slurries. A basic open-loop spray-drying system involves discharging the drying gas into the wider environment at the end of the dryer. However, this drying gas contains a large amount of thermal energy which is removed from the system when the gas is discharged, decreasing the overall energy utilisation of the equipment. Energy conservation and process optimisation are important in industries where large-scale drying processes, such as spray drying, dominate operations [4]. Traditionally, the focus has been on open-loop systems, where significant energy losses occur due to the exhaust of hot drying gases. This study improves on open-loop approaches by focusing on closed-loop systems, a design that inherently recycles energy, making it an ideal candidate for advanced heat- recovery technologies. Closed-loop spray-drying systems present a unique opportunity to significantly improve thermal efficiency by capturing and reusing waste heat through a carefully designed heat-recovery network [5].

Closed loops have been used within superheated steam-drying systems for some time and there is considerable research into them [6]. This is because condensation of the outlet steam is relatively straightforward and recovers the latent heat of vaporisation for water. Superheated steam is also faster during the falling-rate drying period above the inversion temperature [7], where the drying rate decreases as water activity within the material decreases. The higher thermal conductivity and heat capacity of superheated steam leads to higher drying rates for surface moisture above the inversion temperature [8]. To analyse energy consumption more comprehensively in different drying scenarios, the concepts of pinch analysis were developed [9].

Pinch analysis involves identifying the location and magnitude of energy losses in the process, and then using this information to develop a strategy to reduce these losses [10]. It is based on the concept of a “pinch point”, the location in the process where the temperature difference between hot and cold streams is at a minimum. Once this pinch point is calculated, the process conditions are then adjusted to minimise energy losses and to minimise the total hot and cold utilities [11]. It is a key part of process integration, where interactions between unit operations are considered, rather than analysing each unit separately.

Pinch analysis is often used in situations where the hot and cold streams have a large temperature difference, and where heating and cooling are both required in the operation. It has been commonplace in distillation columns due to this feature, but it is suitable for any operation which contains both a heating element and a cooling element [12]. In the case of a closed-loop dryer, the heating element is the hot feed gas, and the cooling element is the condenser loop. By connecting the hot outlet stream to the cold inlet stream with a heat exchanger, overall energy use can be significantly lowered. The hot air leaving the spray dryer can be reused. The recycled heat can also be used to heat the liquid solution before atomisation, reducing the energy requirement for evaporation. Pinch analysis can also help identify opportunities for process optimisation and design changes to be implemented to reduce energy consumption in spray drying [13]. The spray dryer’s design can be altered to improve heat-transfer efficiency between the liquid solution and hot air. For example, it can be used to determine the optimal location and heat of the atomisation nozzle, which can improve the efficiency of the drying process and reduce energy consumption.

For the pinch analysis of the open-loop system, the external temperature often has a significant impact on the pinch analysis of the entire system [14]. In the closed-loop system, any unused thermal energy is returned to the dryer [15]. The closed-loop system is more complex than an open-loop system, so it provides more parameters for adjustment, increasing the optimisation opportunities of the system. For the closed-loop spray-drying system used in this study, cooling the dryer walls by heating recirculating water for the air reheater is beneficial for reducing the deposition of particles on the walls.

The objective of this work was to use pinch analysis to analyse and assess the energy efficiency of a novel closed-loop spray-drying system containing a condenser and reheater. The novelty of the pinch analysis is that it was applied to the analysis of this closed-loop spray-drying system. This system has the advanced aspect of having two interconnected countercurrent loops, one of which is the drying air stream, with the other being the water stream which carries energy back to an air reheater. The air reheater is a new aspect of this closed-loop system that has not been previously reported. Technically, this work assesses the performance of a novel closed-loop spray-drying system containing a condenser and reheater using pinch analysis, providing an accessible and easily used analytical approach to a novel drying system, which is a technically useful advance.

## 2. Materials and Methods

### 2.1. Modelling of Spray Dryer

Following the finer-scale parallel flow/plug flow approach outlined in Langrish (2009) [15], the modelling approach divides the spray dryer into a sequence of control volumes, over which mass and energy balances are performed, and also over which calculations are carried out to estimate heat and mass-transfer rates between the particles or droplets and the gas. The calculation of heat-transfer rates includes estimates of heat losses from the dryer, and the concept of a Characteristic Drying Curve has been used to model the drying kinetics, which are an important part of the mass-transfer calculations.

### 2.2. Experimental Setup

**Figure 1:**
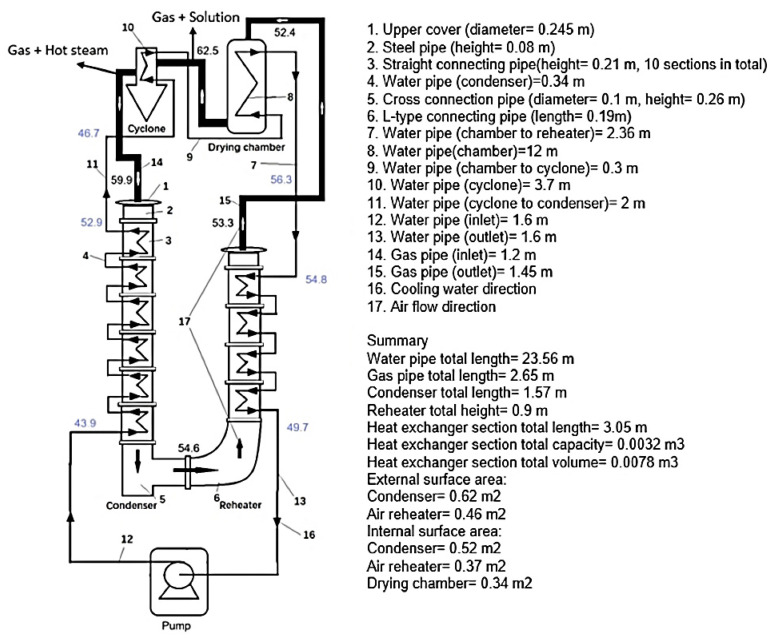
Schematic diagram of multi-section condenser closed-loop drying system. (The temperature values in blue are from Run 13, Table A1, with the gas and cooling water temperatures marked in black and blue, respectively.)

**Figure 2:**
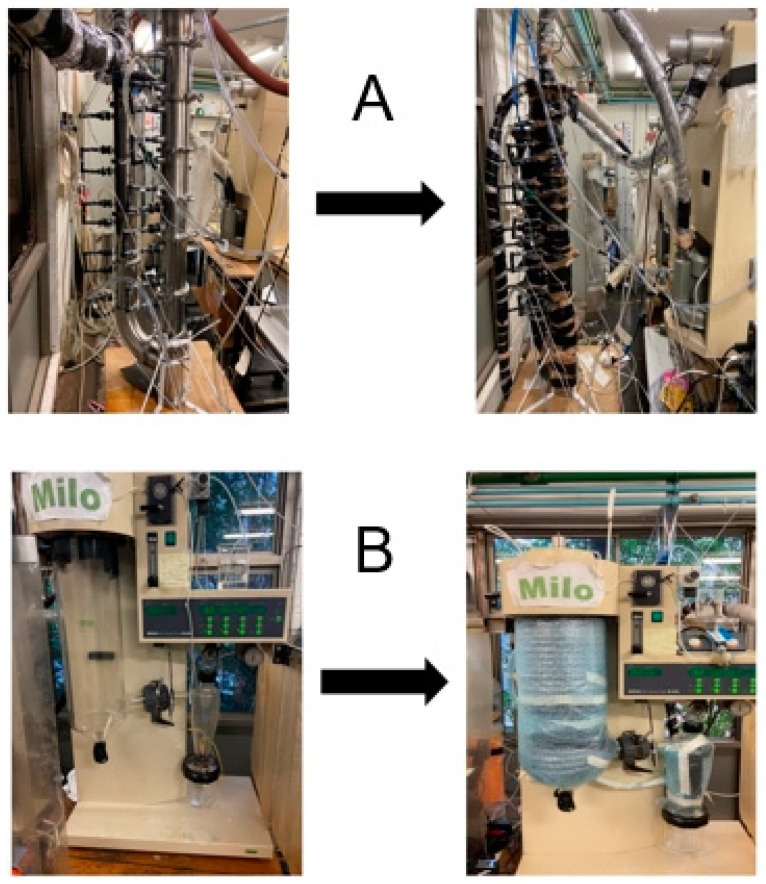
(**A**) Condenser setup of spray dryer; (**B**) Water-fed heat-recycling system [16].

The closed-loop spray-drying system with condenser and air reheater is shown in Figure 1. The air pipes are specially marked, and the remaining connections are condensate pipes. The equipment used is a Buchi B-290 Mini Spray Dryer (Büchi, Switzerland), connected to a condenser and reheater loop. A photograph of the condenser setup is shown in Figure 2A. The solution being sprayed enters at the spray nozzle of the dryer, where it is then sprayed through the spray chamber into the cyclone and separated into its liquid and solid components. The warm, humid air enters the condenser, where the water is condensed and cooled down by a cooling water stream. The condensate liquid is removed after the condenser stage. The cool, drier gas is then sent to an air heater and heated up to the required dryer fluid temperature and used to dry more solution.

Temperature probes were placed at points around the spray dryer, where it was then used until it was found to be at steady state. This was repeated multiple times with different flow rates and system configurations. This was covered in insulation in some later tests. Water was also pumped in vinyl tubing around the dryer and cyclone to recycle heat lost to the environment. This arrangement is displayed photographically in Figure 2B.

### 2.3. Operating Conditions and Parameters

Different flow rates of water were tested by altering the speed of the feed pump located within the bucket water reservoir. Similarly, by adjusting the aspirator and pump rate, the flow of sprayed solution was changed [17]. To determine the ability of the system to run with other solutions, reconstituted powdered milk was also examined. Each set of experiments first pumped water for 30 min to allow the equipment to reach steady state. Parameters which were tested are listed in Table A1.

### 2.4. Preliminary Pinch Analysis Information

**Figure 3:**
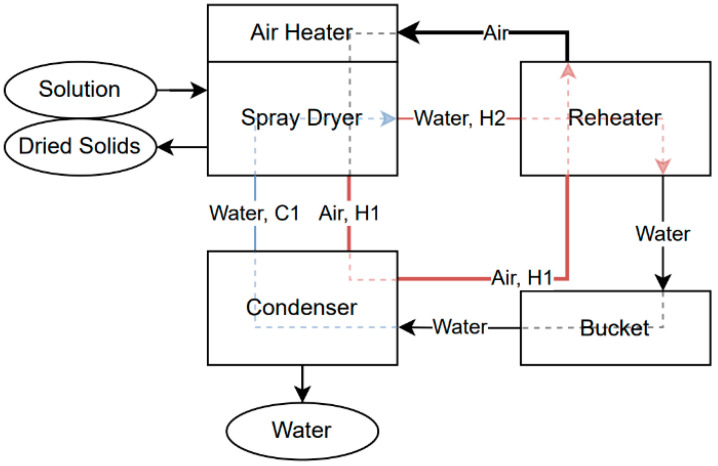
Spray dryer diagram with heat flows demonstrating location of pinch analysis.

The gathered data was further analysed using heat integration and pinch analysis. MATLAB (R2023b) was used to aid in computation, combined with Microsoft Excel, which was also used for the initial data management and temporary graphing. The setup at the time of experimentation is demonstrated diagrammatically in Figure 3, along with the heat flows and hot and cold streams. As can be seen, three streams were located for pinch analysis: two hot and one cold.

## 3. Results

### 3.1. Sample Data and Typical Pinch Analysis

Air and water temperatures were recorded as functions of time with thermocouples placed systematically around the spray-dryer setup. After 30 min of continuous running, the system was found to be at steady state, and the temperatures at designated points were collected. Once enough data were gathered, the system was gradually allowed to relax to ambient temperature. The temperatures during the steady-state period were then averaged to give values for further calculations. An example experiment (Run 1, Table A1) was used to demonstrate the calculation process.

The heat capacity rates were calculated from the mass flow rates of the streams and the known heat capacities of water and of air. This is demonstrated in Equation (1) [18] .

Heat Capacity Rate = specific heat capacity × mass flow rate(1)

CP = c_p_ × m (2)

To determine the total heat duty for each unit operation in a stream, the heat capacity rate was multiplied by the temperature change in the stream. This is explained mathematically in Equation (3) below.

Q = CP × (T_source_ − T_target_) (3)

The inlet and outlet, or source and target temperatures of previously designated hot and cold streams, are also shown below in Table 1, along with the heat capacity rates.

To determine the pinch point, the streams were graphed on a temperature–enthalpy diagram or by using the problem table method [19]. In this instance, the graphical method was used first to visualise the minimum approach temperature, or ΔTmin, clearly.

**Figure 4:**
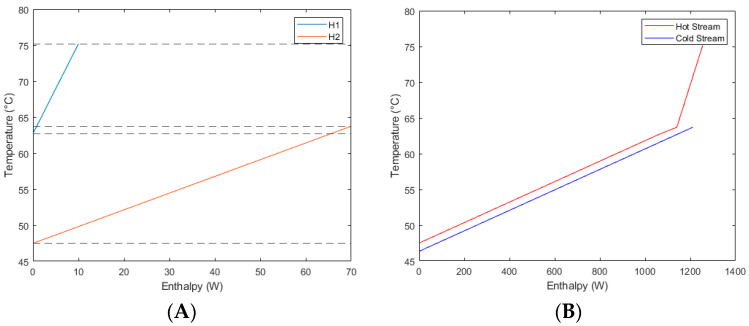
(**A**) Temperature–enthalpy graph for the hot streams. (**B**) Temperature–enthalpy graph of hot and cold streams on a composite curve.

By graphing the known heat loads and temperatures of the hot streams, a hot T/H graph was made. Since there are two streams, a composite curve was created. The initial chart of both hot streams is shown in Figure 4A. The composite curve graph of heat flow can be divided into three parts, and the “dashed lines” are used to mark where the temperature of two heat flows overlaps. The heat load between 75.17 °C and 63.70 °C is represented solely by H1, whilst the heat load between 63.70 °C and 62.72 °C is a combination of both H1 and H2, and the heat load between 62.72 °C and 47.50 °C is solely represented by H2. The heat load was calculated by summing the heat-capacity rates of all streams within the temperature section and is tabulated in Table 2. The heat loads of each section were then determined by multiplying the temperature difference by the total heat capacity rate by again using Equation (3). These values have been added to Table 2. Please refer to the attached Figure-A1 for the temperature–enthalpy graph of all experimental data.

The pinch point and corresponding approach temperature were determined using Figure 4B. The closest approach between the hot and cold stream, the pinch temperature, occurred when the hot stream was at a temperature of 63.7 °C. By calculating the vertical distance from this point and the cold stream, the minimum approach temperature was calculated. In this instance, the ΔTmin was determined to be 1.0075 °C. As can be seen, the minimum approach temperature was very low, which indicates efficient heat exchange between the hot and cold streams. In this situation, there was no need for additional heat exchangers, as the system was already very efficient. The heat exchangers, which are already present in the form of the condenser and the reheater, could be decreased in size if required for cost savings, but this is not necessary.

**Figure 5:**
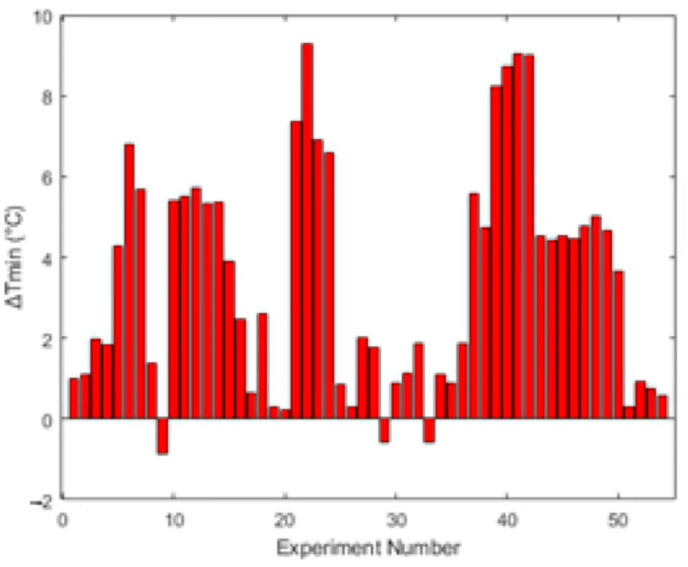
ΔTmin values for all experiments.

As previously mentioned, the minimum approach temperature works as a good metric for the efficiency of heat transfer between the hot and cold streams and therefore the effectivity of the heat-exchange networks within the system. By calculating and comparing the values of ΔTmin for many runs, assessments to find the most sensitive parameters can be performed. A series of experiments testing the values noted in Table 2 were completed and the data collated. These data were analysed with the same methods as the sample calculation previously, and the results are shown in the figures below. The range of calculated ΔTmin values is shown graphically in Figure 5. There are a few values of ΔTmin that are negative, indicating a ‘cold’ stream with a higher temperature than that of the corresponding ‘hot’ stream (Run 9, 29, 33 in Table A1).

The only commonality between these runs is that the cooling water flow rate was at the lowest tested value. A possible reason is that, when the humid air in the system undergoes phase change (condensation or evaporation), additional energy is released, raising the temperature of the “cold” stream above that of the “hot” stream, particularly when the approach temperatures are low [20]. An average approach temperature, ΔTmin, of 3.48 K, was calculated. This is very low, indicating a heat-transfer rate which is near the maximum physical limit.

### 3.2. Pinch Analysis for All Data and Approach Temperatures

**Figure 6:**
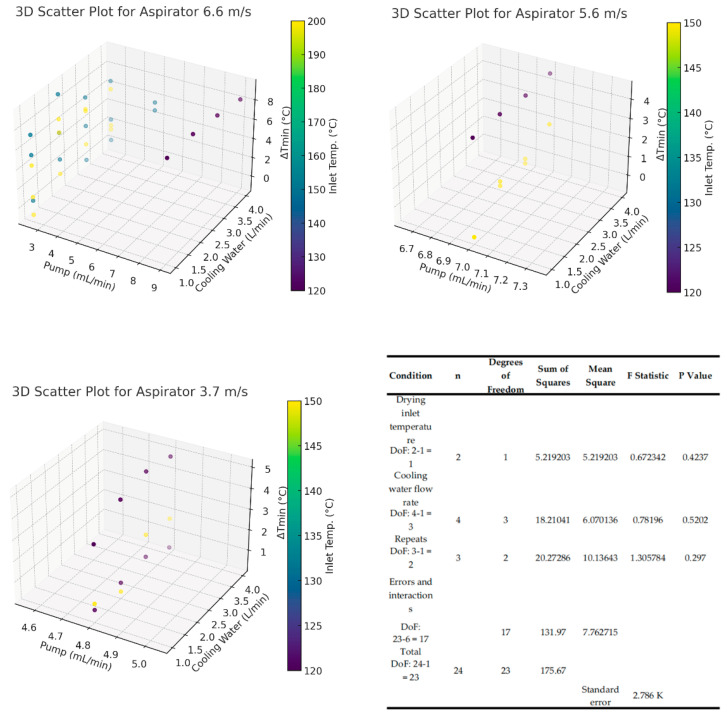
Impact of pump rate, cooling water flow, and inlet temperature on ΔTmin across different aspirators (3.7, 5.6, 6.6 m/s) and its ANOVA results.

The initial analysis, based on a visual inspection of the 3D scatter plots (Figure 6), suggested that there may be interactions between variables such as pump rate, cooling water flow rate, and the inlet temperature on ΔTmin. For example, the pump rate and cooling-water flow rate significantly influence ΔTmin. As the pump rate increases, ΔTmin shows a general increasing trend. The inlet temperature (°C), represented by the colour gradient, also plays an important role in influencing ΔTmin. Higher inlet temperatures tend to correlate with higher ΔTmin values, suggesting that the inlet temperature strongly affects the heat-transfer efficiency within the system. At different aspirator values, which are the main air flow rates through the dryer, the interactions between these variables differ. At the higher aspirator rate of 6.6 m/s, the effects of pump rate and cooling water flow on ΔTmin become more complex, indicating that the increased airflow may introduce non-linear effects on the overall heat-transfer processes.

However, after conducting the ANOVA analysis, there was no statistically significant effect of any individual variable on ΔTmin, which aligns with the subjective review of the approach temperatures. It should also be noted that all approach temperatures are very low, with all values below 10 K, indicating very good heat recovery [21]. The standard error for the temperature data is also shown to be approximately 2.7 K, which further reinforces the suggestion that the approach temperatures are near zero, as many values are smaller than the calculated standard error. In these cases, the approach temperatures are likely to be very close to zero, indicating that the heat-transfer efficiencies were close to the physical limits for heat recovery. This situation may also partially explain the negative approach temperature values seen in some of the runs, as their actual approach temperature might just have been very low, and the uncertainty in the temperature measurements might have resulted in an apparently negative approach temperature. Heat transfer could be increased further but only with the input of additional mechanical energy, such as vapour recompression [22], although research into the feasibility of such a system is outside of the scope of this analysis.

These results again indicate that the improvement potential of the heat-recovery system created around the spray-dryer system is very small, as the heat recovery is near or at the limit, as shown by the low approach temperatures. As mentioned previously, it may be possible to decrease the number of stages within the condenser and reheater while keeping a similar rate of heat recovery. This is due to the ratio between the surface area, the temperature differences, and the heat-transfer rates within a heat exchanger not being linear, especially when close to the maximum transfer rates [5]. Further research into the economic feasibility will be required, as the removal of stages will result in a lower capital cost and a decrease in the required space for the equipment. This is a future extension of this research.

It can also be seen that as the *p*-values for all results are much higher than 0.05, the null hypothesis of no significant effects of operating conditions on the approach temperatures should be accepted statistically. The 3D plots served as an initial exploration tool, offering visual insight into potential trends, but the statistical evidence from ANOVA confirms that these interactions were not significant. This suggests that the system’s performance is not significantly reliant on the variations in drying parameters, and that the heat-recovery system is operating near its physical limit, requiring additional mechanical energy (such as vapour recompression) to achieve further improvements. As a result, the lack of a significant relationship between input parameters and ΔTmin points to a broader applicability of the heat-recovery network, which could function across different systems and scales without being sensitive to specific operating conditions.

## 4. Discussion

### 4.1. Approach Temperature and Heat-Transfer Performance

The minimum approach temperature found here of 3.48 K (under 4 K) may be compared with the literature on pinch analysis as applied to other drying systems. Kemp [20] suggests using a minimum approach temperature difference of 20 K for the pinch analysis of drying systems in general. Studying an alcohol distillery with pulp drying, Ficarella and Laforgia [23] used a minimum approach temperature difference of 10 K. Our values of under 4 K for the minimum approach temperature are comparable with those of Harkin et al. [24], who studied a power plant involving the pre-drying of coal. They suggested a minimum approach temperature difference of 20 K, and they stated that a difference of 3 K was optimistic. The closest analysis in the literature to this study is that by Patel and Bade [5], who studied a spray dryer and heat-recovery system with a minimum approach temperature of 10 K. Kaviani et al. [25] studied a dairy factory involving milk powder production and a spray dryer, using a minimum approach temperature of 50 K. The minimum approach temperature found in this study is therefore low by the standards of the literature.

Several cases of negative approach temperatures were also observed, which might have resulted from the phase changes within the system, such as condensation, where latent heat is released, increasing the temperature of the cold stream beyond that of the hot stream. These anomalies emphasise the complexity of accurately predicting heat-transfer behaviour in highly efficient systems. The data collected consistently highlight the stability of the heat-recovery system in this spray-drying setup. The low approach temperatures achieved suggest that the heat exchangers, including the condenser and reheater, were operating at high efficiency. More importantly, the stability of ΔTmin across various experimental conditions demonstrates that the system can maintain effective heat recovery despite fluctuations in operational parameters. This indicates that the system can be applied across different scales without the need for significant changes to its configuration.

### 4.2. Comparison with Existing Studies

Pinch technology has already been applied to open-loop drying systems by Kemp [20], but this open-loop system effectively recaptures and reuses energy in a way that is novel and has not been reported before, particularly by using the air reheater to recover energy from the condenser. The approach temperatures used in these open-loop studies are considerably greater (20 K) than those found in this experimental study for the integrated closed-loop system (under 4 K), indicating significantly better thermal integration in this closed-loop system compared with many open-loop systems. Other studies of energy efficiency on open-loop systems (Sarker et al. [26]; Surendhhar et al. [27]) have confirmed their low thermal efficiencies.

This closed-loop drying system has novelty in that it includes an air reheater to recover energy from the condenser. Drying with superheated steam is another way to recover energy from drying systems (for example, Guo et al. [28]), but the need to address the pressurisation requirements of superheated steam drying is a disincentive to use these systems. The system described in this paper is a normal air-water vapour system, with the unique features of including a closed loop with both a condenser and reheater.

Another approach to the analysis of this drying system is a full energy and exergy analysis (for example, Aghbashlo et al. [29]; Amjad et al. [30]; Dincer and Rosen [31]; Erbay and Hepbashli [32]; Johnson and Langrish [33]; Lei and Langrish [16]), which is a more comprehensive, complex, and more difficult analytical technique. This approach has the advantage of including non-thermal components of energy availability, such as pressure. However, the data collection and analysis requirements are more challenging, and pinch analysis, as used in this work, offers potential advantages in ease-of-use for operational engineering purposes.

Another advantage of a closed system is a higher recirculation rate. Golman and Julklang [34] found, in their study of open-loop systems, that increasing the recirculation ratio of exhaust gas can improve the energy efficiency of spray-drying equipment. A closed system can be considered as an infinite loop system. In addition to improving the recirculation ratio, our closed-loop system goes a step further by integrating air reheaters. This approach results in even lower approach temperatures (ΔTmin), suggesting superior heat-recovery performance.

### 4.3. Future Research Suggestions and Prospects for Practical Applications

However, this study also encountered limitations, such as phase changes and extreme temperature conditions that were not fully accounted for in the experimental design. These factors could introduce complexities that influence heat-transfer behaviour. For the purposes of this analysis, however, the primary focus was on evaluating the overall heat-recovery potential, which remains consistent across various operational configurations. As future research expands to larger-scale systems, these complexities will need to be considered in greater detail, particularly in industrial-scale applications where heat- transfer dynamics may differ.

Some valuable future work could include testing different configurations of condensers and reheaters, as well as exploring the use of steam recompression to enhance heat-transfer rates [35]. Furthermore, the scalability of this system should be explored through larger-scale tests to verify that the energy-recovery performance remains consistent when applied to industrial-scale equipment.

## 5. Conclusions

It can be seen from the results of ANOVA tests that there is no significant effect of any variable on the approach temperature. The consistently small approach temperatures (all below 10 K, with an average of 3.48 K) suggest excellent heat recovery. In several instances these approach temperatures are very close to zero, demonstrating the attainment of the physical limit for heat transfer and recovery. This is further supported by the standard error of 2.7 K, indicating that the minimum approach temperatures may be lower than are calculated, indicating possibly an even better heat-transfer rate. The presence of negative approach temperature values in some runs is most likely a result of very low approach temperatures combined with unaccounted-for phase transitions in the system.

The results collected unequivocally indicate the value of integrating a heat-recovery system into spray-drying equipment. The low approach temperature denotes a high rate of heat transfer, which stayed relatively constant over different testing parameters. There is clearly more room for experimentation and research in this area, such as testing the sizing of and number of stages of the condenser and reheater and the addition of vapour recompression to further increase the heat-transfer rate. The lack of relationship between any input parameters and result denotes that the heat-recovery network is not dependent on one particular variable and can most likely be transferred to other spray-drying systems at different scales. Scale-up tests on larger pieces of equipment with a greater powder production capability must be performed to confirm these hypotheses.

## Figures and Tables

**Table 1:** Thermodynamic information of designated streams.

**Stream**	**Source Temperature (°C)**	**Target Temperature (°C)**	**Heat Capacity Rate (W/K)**
H1	75.17	62.72	10
H2	63.70	47.50	69.77
C1	46.34	63.70	69.77

**Table 2:** Heat capacity rates and heat loads of hot-stream sections.

Interval (°C)	Temperature Difference	Total Heat Cap Rate (W/K)	Heat Load (W)
75.17–63.70	11.48	10	114.76
63.70–62.72	0.9748	79.77	77.75
62.72–47.50	15.23	69.77	1062.34

**Figure A1:**
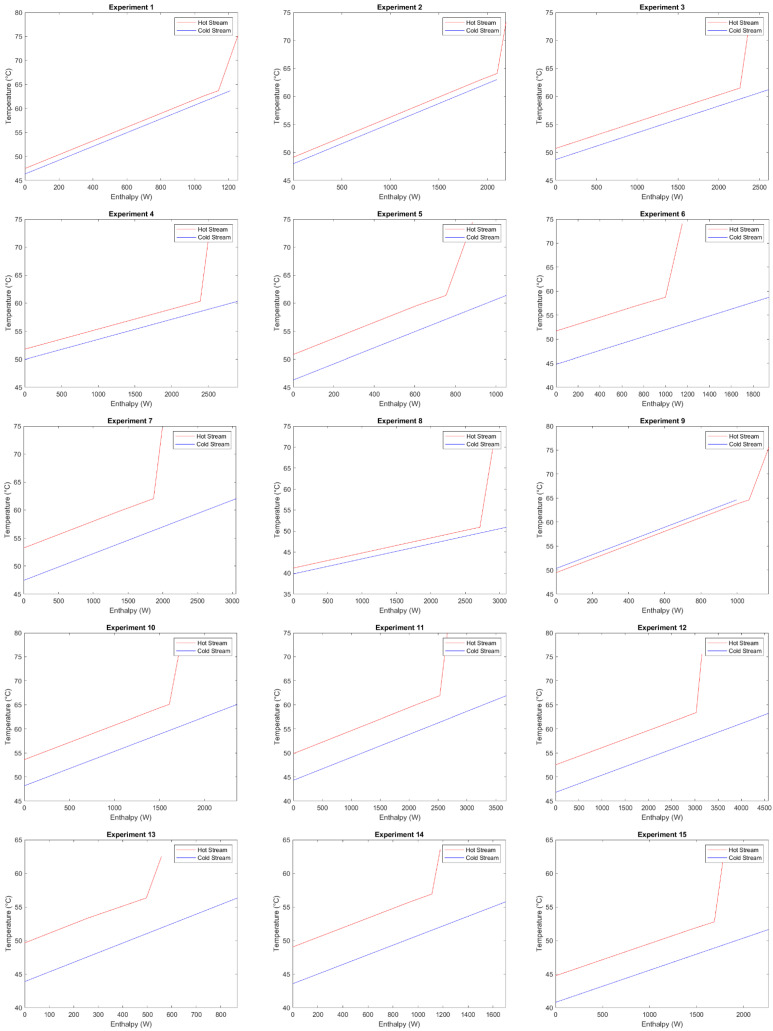

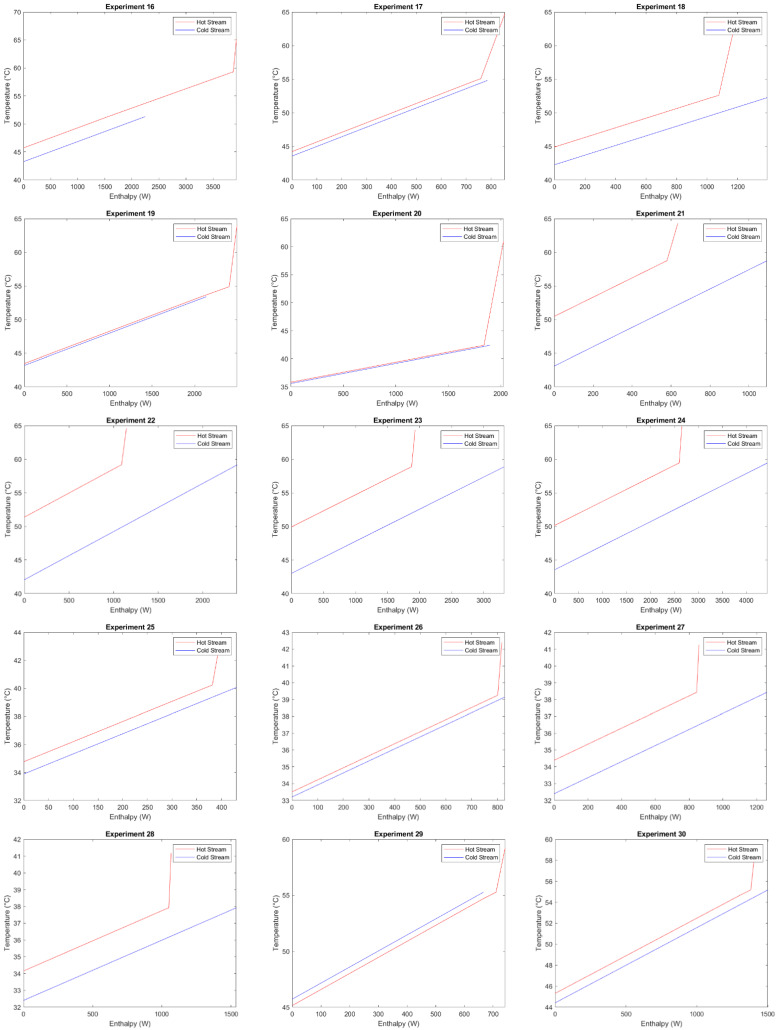

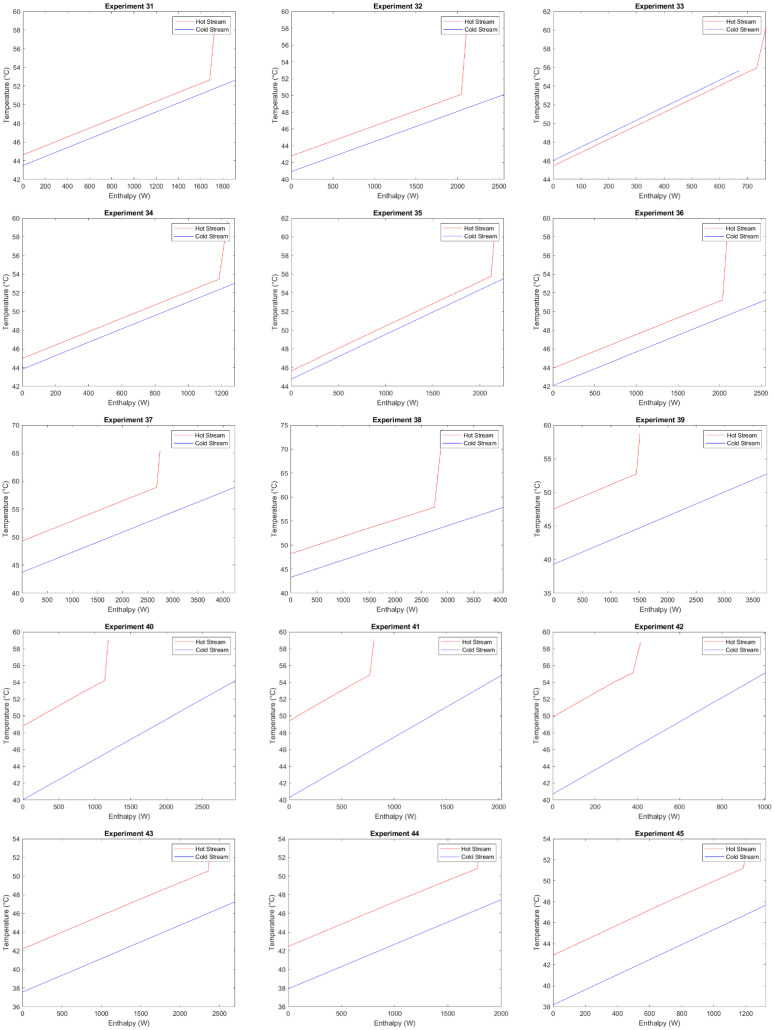

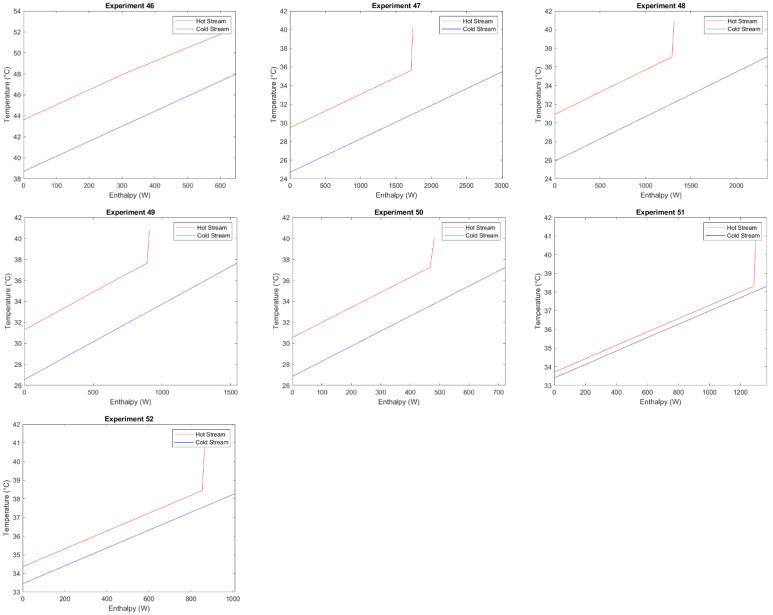
Experimental pinch analysis graphs.

## Data Availability

The original contributions presented in the study are included in the article. Further inquiries can be directed to the corresponding author.

## Appendix A

**Table A1:** Full dataset used for pinch analysis.

Run	Aspirator m/s	Pump Flow mL/min	Water Flow L/min	Spray Dryer °C	Dryer Out °C	Condenser in (Dry) °C	Condenser In (Wet) °C	Condenser Out (Dry) °C	Condenser Out (Wet) °C	Reheater out (Dry) °C	Reheater out (Wet) °C	Dryer Return °C	Water Condenser in °C	Water Condenser Out °C	Water Cyclone in °C	Water Cyclone Out °C	Reheater in °C	Reheater Out °C	Ambient °C	Solid Conc.
1	6.6	2.5	1	200	75.1716	71.5650	69.5969	63.2492	60.7077	62.7212	59.8997	59.7385	46.3419	48.7322	46.8536	63.6960	61.3804	47.4942	23	0
2	6.6	2.5	2	200	73.2676	71.0983	70.0348	65.2042	60.9506	64.1115	56.0566	62.8647	47.9838	51.5853	50.2930	63.0232	62.6006	49.1362	23	0
3	6.6	2.5	3	200	73.0557	70.0414	69.2088	62.4383	58.5395	61.4970	54.7905	55.0064	48.7362	53.3926	51.2943	61.2199	60.8099	50.7123	23	0
4	6.6	2.5	4	200	73.7805	69.5490	68.4137	61.6641	60.1421	60.1850	58.5497	57.1121	49.9603	54.1536	52.8001	60.3432	59.1038	51.7962	23	0
5	6.6	2.5	1	200	74.4379	72.7546	66.2832	61.9347	60.5871	59.5908	53.6764	53.8572	46.3158	52.0430	49.4764	61.3903	61.1379	50.8397	23	0
6	6.6	2.5	2	200	74.0747	72.5767	65.4560	59.8542	58.4660	57.0368	51.3401	45.7998	44.7938	51.1428	49.2957	58.7424	55.5523	51.7058	23	0
7	6.6	2.5	3	200	74.5935	72.9138	66.3253	62.4347	60.9557	59.9065	54.2300	48.2158	47.4468	53.9761	52.8337	62.0548	60.6574	53.2377	23	0
8	6.6	2.5	4	200	72.4710	70.8197	68.3685	53.1593	51.6917	50.4036	45.9238	41.4590	39.8112	45.5723	44.1722	50.9115	51.8605	41.2088	23	0
9	6.6	2.5	1	200	75.6280	72.8485	68.3470	64.5862	56.2058	63.7678	54.1171	62.7215	50.3387	53.9736	51.8090	64.6282	63.3372	49.4757	23	0
10	6.6	2.5	2	200	75.9942	73.2409	68.7054	65.1826	56.7303	64.3810	56.4642	63.3208	48.1746	54.3728	52.4255	65.0997	63.4728	53.6270	23	0
11	6.6	2.5	3	200	74.9366	72.4010	67.3160	62.0967	54.1391	60.4718	52.3614	60.2352	44.3232	53.9788	53.1835	61.9069	61.2913	49.8882	23	0
12	6.6	2.5	4	200	75.5303	72.5411	68.6941	64.4329	57.1152	63.3840	55.7056	62.0126	46.8143	53.0367	51.0812	63.2462	63.5067	52.5463	23	0
13	6.6	2.5	1	150	62.4818	59.8986	58.4787	54.5915	52.7616	53.3468	51.7184	52.3748	43.9004	52.8560	46.7391	56.3396	54.8420	49.6587	23	0
14	6.6	2.5	2	150	63.5980	60.9155	60.1536	57.3471	55.0795	56.9267	51.8611	56.4997	43.5770	52.3766	46.4599	55.7672	54.2420	49.0446	23	0
15	6.6	2.5	3	150	62.3273	59.9560	58.2200	53.7477	50.7739	52.7936	49.9203	52.3815	40.8085	46.3682	44.8120	51.6500	50.2093	44.7691	23	0
16	6.6	2.5	4	150	65.1282	62.5910	62.5837	61.1674	59.9958	59.3121	59.0479	57.2354	43.2633	46.4472	45.3751	51.2969	50.3529	45.7208	23	0
17	6.6	2.5	1	150	64.7488	63.0570	59.0870	56.3066	49.4585	55.0577	47.0135	54.2013	43.5524	46.6839	44.7374	54.8074	55.0495	44.2170	23	0
18	6.6	2.5	2	150	63.7057	62.3226	58.1949	53.7238	47.1978	52.5861	43.0198	50.2714	42.2570	46.3626	45.0676	52.2475	51.6151	44.8941	23	0
19	6.6	2.5	3	150	63.9824	62.7074	53.5206	58.6963	50.8856	54.8860	48.6885	54.1493	43.1828	47.0230	46.2847	53.3903	51.2280	43.4638	23	0
20	6.6	2.5	4	150	60.9944	59.8292	55.6201	43.6803	38.0877	41.9691	35.8439	41.0740	35.5947	38.5845	37.6436	42.3927	42.1432	35.8157	23	0
21	6.6	2.5	1	150	64.3330	62.1136	60.3219	60.1368	52.4636	58.6439	50.8940	58.0908	43.0850	52.0906	49.5797	58.7382	58.7667	50.4560	23	0
22	6.6	2.5	2	150	64.5907	62.0912	60.4948	59.8218	52.0853	58.8620	51.1193	58.0709	42.0560	49.5240	49.4595	59.1631	58.4987	51.3731	23	0
23	6.6	2.5	3	150	64.3372	61.9280	60.3121	59.4930	52.2952	58.5138	51.3354	57.8387	43.0070	49.3454	49.1745	58.8703	57.8698	49.9273	23	0
24	6.6	2.5	4	150	64.8557	62.3371	60.6978	59.9265	53.0794	59.1275	52.2994	58.2776	43.5429	49.8127	49.0696	59.4424	58.5646	50.1417	23	0
25	3.7	4.8	1	150	43.0610	42.0934	40.3255	40.1900	38.1705	40.2316	37.5786	39.0654	33.8959	37.1251	35.1198	40.0699	38.0944	34.7726	23	0
26	3.7	4.8	2	150	42.4005	41.4332	39.6324	39.5595	38.1670	39.2535	37.3231	39.3341	33.2073	34.7687	34.3833	39.1461	37.4963	33.5128	23	0
27	3.7	4.8	3	150	41.2514	40.1174	38.5030	38.2831	37.3100	38.3046	36.0507	37.1052	32.4036	34.2982	33.3714	38.4294	37.0754	34.3952	23	0
28	3.7	4.8	4	150	41.1735	40.0006	38.2505	37.9444	36.0789	37.8402	35.8396	37.2014	32.4063	34.9469	33.7657	37.9237	36.2974	34.1599	23	0
29	5.6	7	1	150	59.2081	57.4708	59.3151	54.4179	53.6905	54.7271	51.8439	53.1262	45.7232	48.2874	47.4704	55.2689	51.1556	45.1504	23	0
30	5.6	7	2	150	59.3290	57.1404	59.5271	55.7261	53.5567	54.6225	51.7653	52.5403	44.4190	48.1995	47.3811	55.1818	50.5180	45.3199	23	0
31	5.6	7	3	150	58.6593	57.3873	58.0530	53.2880	51.1264	52.3019	49.7926	50.3541	43.4999	46.1538	45.4126	52.6616	48.6706	44.6377	23	0
32	5.6	7	4	150	58.0039	56.7561	56.4113	51.0477	48.7600	49.9113	46.8551	48.7583	40.9359	44.2051	43.3998	50.1217	47.0328	42.8005	23	0
33	5.6	7	1	150	60.2479	60.0823	58.6729	55.5879	54.8087	55.9241	52.9660	54.3096	46.0346	48.5657	47.9026	55.6471	52.4247	45.4455	23	0
34	5.6	7	2	150	59.7842	59.1857	58.5144	54.4414	52.2584	53.4521	50.9325	51.5064	43.8380	46.5052	45.7740	53.0387	49.0197	44.9693	23	0
35	5.6	7	3	150	60.4843	60.6462	58.2358	56.8068	54.6468	55.7730	52.9022	53.6931	44.7731	48.5299	47.7378	55.5464	50.8544	45.6664	23	0
36	5.6	7	4	150	59.1500	57.8984	57.5606	52.1893	49.9024	51.0592	48.0026	49.9234	42.0854	45.3556	44.6207	51.2414	48.1909	43.9458	23	0
37	6.6	4.8	4	150	65.5202	63.5964	60.2444	59.4349	52.7282	58.5134	51.9039	57.5938	43.7100	49.3801	49.3509	58.8914	57.5729	49.2975	23	0.3
38	6.6	4.8	4	150	74.1482	70.8732	64.9347	55.3062	51.8788	52.9377	47.3341	52.6062	43.2956	48.5530	45.6755	57.8701	56.6894	48.2075	23	0.5
39	6.6	9	4	120	58.7008	55.3529	54.1977	52.1243	50.9580	52.1098	51.7835	49.2899	39.3090	44.8927	44.6517	52.7253	51.4744	47.5482	23	0
40	6.6	9	3	120	58.9906	56.5364	55.5239	54.1905	52.3673	53.2020	50.6812	52.9003	40.0378	45.9908	45.7216	54.2073	53.0120	48.8041	23	0
41	6.6	9	2	120	59.0826	57.0488	56.1272	54.8524	52.9781	53.7834	50.9993	53.0240	40.2975	46.7175	46.3749	54.8557	53.5224	49.4213	23	0
42	6.6	9	1	120	58.8236	57.3330	56.5013	55.2390	53.4838	53.9600	51.2352	52.7683	40.7103	46.7482	45.0560	55.1297	54.2785	49.8648	23	0
43	5.6	7	4	120	52.8293	52.5631	51.9299	50.8960	50.1603	50.5494	49.7287	49.2378	37.5746	44.2184	43.5480	47.2485	47.2162	42.1930	23	0
44	5.6	7	3	120	53.1210	52.6973	52.0626	51.0922	49.8989	50.8235	49.4071	50.3030	37.9161	44.4559	43.8870	47.4720	46.6733	42.4596	23	0
45	5.6	7	2	120	53.1719	52.8854	52.3538	51.3648	50.6249	51.2039	49.7381	50.2266	38.1805	44.2317	43.2023	47.6719	46.8098	42.9117	23	0
46	5.6	7	1	120	53.4273	53.5152	52.9259	52.0160	51.2489	51.9207	50.8454	50.3537	38.7065	45.2893	44.6410	47.9875	47.9033	43.6178	23	0
47	3.7	4.8	4	120	40.2615	39.0251	36.0683	37.7155	34.6997	35.6237	31.2515	35.3360	24.6923	32.6744	30.4618	35.5018	34.1155	29.4740	23	0
48	3.7	4.8	3	120	40.9498	39.6639	38.4730	37.3453	33.5291	37.0186	32.8360	36.7503	25.8874	32.1705	30.0846	37.0857	35.4825	30.9051	23	0
49	3.7	4.8	2	120	40.7862	39.4800	38.3624	37.4973	33.5539	36.3201	32.2930	36.5932	26.5587	32.2572	29.9923	37.6397	36.0107	31.2878	23	0
50	3.7	4.8	1	120	40.0784	38.7690	37.7959	37.3102	33.5756	36.8201	32.1513	36.0919	26.8681	33.4480	31.6668	37.2357	35.5271	30.5587	23	0
51	3.7	4.8	4	120	41.7119	40.4313	38.6167	38.7246	37.2602	38.1673	34.2688	37.0507	33.4071	36.2079	34.4072	38.3118	37.5670	33.7110	23	0
52	3.7	4.8	3	120	41.6239	40.2819	38.8122	38.4959	37.3783	38.4377	36.9453	37.1426	33.4491	36.4336	34.4005	38.2860	37.6939	34.3605	23	0
53	3.7	4.8	2	120	41.4106	39.9888	38.7140	38.3102	37.4186	38.4001	37.1410	36.9191	33.5626	36.4908	34.7835	38.2930	37.6616	34.3045	23	0
54	3.7	4.8	1	120	40.6386	39.2694	38.4597	38.1633	37.1657	37.7989	36.7037	36.4290	33.2502	36.3121	34.3388	38.0856	37.4039	33.8363	23	0
